# Rochester Healthy Community Partnership: Then and now

**DOI:** 10.3389/fpubh.2022.1090131

**Published:** 2023-01-10

**Authors:** Mark L. Wieland, Jane W. Njeru, Jennifer A. Weis, Abby Lohr, Julie A. Nigon, Miriam Goodson, Ahmed Osman, Luz Molina, Yahye Ahmed, Graciela Porraz Capetillo, Omar Nur, Irene G. Sia

**Affiliations:** ^1^Division of Community Internal Medicine, Geriatrics, and Palliative Care, Mayo Clinic, Rochester, MN, United States; ^2^Rochester Healthy Community Partnership, Rochester, MN, United States; ^3^Center for Clinical and Translational Science, Mayo Clinic, Rochester, MN, United States; ^4^Hawthorne Education Center, Rochester, MN, United States; ^5^Alliance of Chicanos, Hispanics, and Latin Americans, Rochester, MN, United States; ^6^Intercultural Mutual Assistance Association, Rochester, MN, United States; ^7^Division of Public Health, Infectious Diseases, and Occupational Medicine, Mayo Clinic, Rochester, MN, United States; ^8^Somali American Social Service Association, Rochester, MN, United States

**Keywords:** community-based participatory research, sustainability, immigrant health, health equity, health promotion

## Abstract

Community-engaged research partnerships promote health equity through incorporation of regional contexts to inform partnership dynamics that shape research and interventions that reflect community voice and priorities. Long-term partnerships build trusted relationships and promote capacity building among community and academic partners, but there are many structural barriers to sustaining long-term partnerships. Here we describe lessons learned from sustaining Rochester Healthy Community Partnership (RHCP), an 18-year community-based participatory research (CBPR) partnership in Southeast Minnesota. RHCP collaborates with immigrant and refugee populations to co-create interventions that promote health equity for community health priorities. Challenges to sustainability include a tension between project-based funding and the needs of long-term community-based research infrastructure. These challenges can be met with a focus on shared CBPR principles, operating norms, partnership dynamics, and governance. RHCP began in 2004 through identification of a community health priority, defining the community, and establishment of CBPR principles. It grew through identification of broader community health priorities, capacity building for community and academic partners, and integration of diverse learners. We describe the capacity for RHCP to respond to new societal contexts, the importance of partnership dynamics as a barometer for partnership health, and lessons learned about sustainability of the CBPR partnership.

## Introduction

In 2004, a community-academic partnership developed between Mayo Clinic and an adult education center that serves new immigrants and refugees. Rochester Healthy Community Partnership (RHCP) matured by formalizing operating norms, adopting community based participatory research (CBPR) principles, and adding partners from multiple sectors. RHCP has developed an effective community-based research infrastructure that has facilitated extensive research training for community partners. RHCP community and academic partners have co-created several initiatives that addressed community priorities and contexts (Table 1). RHCP has adapted an empirically derived CBPR conceptual model through in-depth evaluation. Community and academic partners jointly conduct every phase of research including disseminating results, implementing sustainability plans, and co-authoring scientific products.

**Table 1:** Examples of RHCP initiatives.

**Title**	**Description**	**Funding**	**Outcomes**
Let's talk about TB	**Background:** High incidence of tuberculosis (TB) in Olmsted County, disproportionately affecting refugees **Approach:** Opened a community-wide dialogue around the issue; Described perceptions of TB and its prevention among recent immigrants and refugees	National Institute of Allergy and Infectious Diseases (R03), 2008–2011	Defined prevalence of TB and established an effective community-owned process for screening at an adult education center (1). Sustainably changed TB screening policy for at-risk populations (2).
Healthy immigrant families	**Background:** There is a steep accumulation of cardiovascular risk after immigration **Approach:** Community-derived family-focused culturally-appropriate intervention to improve dietary quality and physical activity among immigrant and refugee families (randomized trial)	National Heart, Lung, and Blood Institute (R01), 2011–2018	Improved dietary quality but not physical activity at 12 months (sustained at 24 months) (3, 4).
Healthy immigrant community	**Background:** There is a steep accumulation of cardiovascular risk after immigration **Approach:** Assess the efficacy of a social network-informed CBPR-derived health promotion intervention on measures of cardiovascular risk in two immigrant communities through this process: Social network analysis → intervention development → pilot test intervention → cluster randomized trial	National Institute on Minority Health and Health Disparities (P50, embedded R01-level project), 2021–2026	Social network analysis with Somali and Latinx communities completed (5, 6). Pilot of the intervention showed reduction of cardiovascular risk (7). Cluster randomized trial is underway.
Club fit	**Background:** Higher rates of overweight among children from low-income households **Approach:** Multi-component healthy eating and activity intervention (policy and practice) at a Boys & Girls Club	Mayo Clinic, 2014–2016	Improved motivation and confidence for healthful behaviors among at-risk youth (8).
Stories for change: diabetes	**Background:** Diabetes has been a RHCP community concern for many years and disparities are significant among Somali and Latinx groups **Approach:** Co-creation of a digital storytelling intervention for diabetes self-management	National Institute of Diabetes and Digestive and Kidney Diseases (R01), 2018–2023	Improved glycemic control among participants who viewed the digital storytelling intervention (9, 10). Randomized trial of efficacy is near completion.
Closing the gap: reduction of cancer prevention disparities	**Background:** People with limited English proficiency (LEP) receive fewer recommended preventive cancer screenings than English-speaking patients, leading to detection of disease at later stages and higher disease-related death than patients who speak English well **Approach:** RHCP-clinic collaboration to open community dialogue; develop and test clinic and community-based interventions	Mayo Clinic, 2018–2023	Pilot test of clinic-based intervention underway (11). Digital storytelling intervention developed with Latinx participants for colorectal, breast, and cervical cancer screening.
COVID-19 community-engaged crisis and emergency risk communication	**Background:** Data emerged around COVID-19 health disparities in early 2020. Credible COVID-19 messages were not reaching immigrant communities with limited English proficiency **Approach:** RHCP developed a community-engaged bidirectional risk communication framework to disseminate COVID-19 information and inform policy makers	Mayo Clinic, 2020–2022	Pilot and implementation studies have demonstrated feasibility, acceptability, reach, 18-month sustainability, scalability and perceived effectiveness of a bidirectional COVID-19 CERC intervention across multiple groups disproportionately affected by the pandemic (12, 13).

In this manuscript, we describe the mechanics of starting and sustaining a longitudinal CBPR partnership as experienced by RHCP community and academic partners over the last 18 years. We describe the tension between the biomedical emphasis of funders and the social structure of participatory work and implications for partnership infrastructure. We describe lessons learned about partnership dynamics in the course of conducting specific RHCP projects and responding to specific societal and regional contexts.

### RHCP then: Lessons learned from starting a CBPR partnership

#### Identification of a community health priority and definition of community

Rochester Healthy Community Partnership (RHCP) started in 2004 as a partnership between clinician-researchers at Mayo Clinic in Rochester, an academic medical center in Minnesota, and Hawthorne Education Center (HEC), an adult education center within Rochester Public Schools serving diverse immigrant and refugee communities in Rochester, Minnesota. The impetus for the partnership arose from HEC's concern for tuberculosis (TB) among its learners and in an effort to understand why an established TB prevention and control program was ineffective among its learners. Several cases of active TB had been diagnosed among learners, prompting an environment of fear and TB-related stigma. Previous attempts at voluntary TB screening had very low participation. HEC staff (JAN) approached Mayo Clinic with this concern through a volunteer (JAW), who connected with a TB physician specialist (IGS). Additional HEC and Mayo Clinic staff were engaged, and it became clear that another top-down approach was unlikely to be successful. The team collaboratively explored targeted TB evaluation and developed innovative ways of effective communication of health information, while at the same time, building community trust and capacity to participate in the research process. The team recognized that this approach aligned with CBPR principles (14). Community and academic partners took CBPR coursework together, and this process planted the seed for what would later become RHCP.

Utilizing a CBPR approach, this community-academic team discovered several factors related to knowledge and perceptions of TB, which contributed to avoidance of discussing TB, and unwillingness to participate in screening (15). This led the team to design a community-led TB education and screening program which was implemented at HEC. The program was successful in terms of educating learners and staff and improving screening and treatment rates (1). The program was subsequently incorporated into ongoing HEC processes and has been sustained for several years (2).

#### Adoption of CBPR principles and operating norms

**Figure 1:**
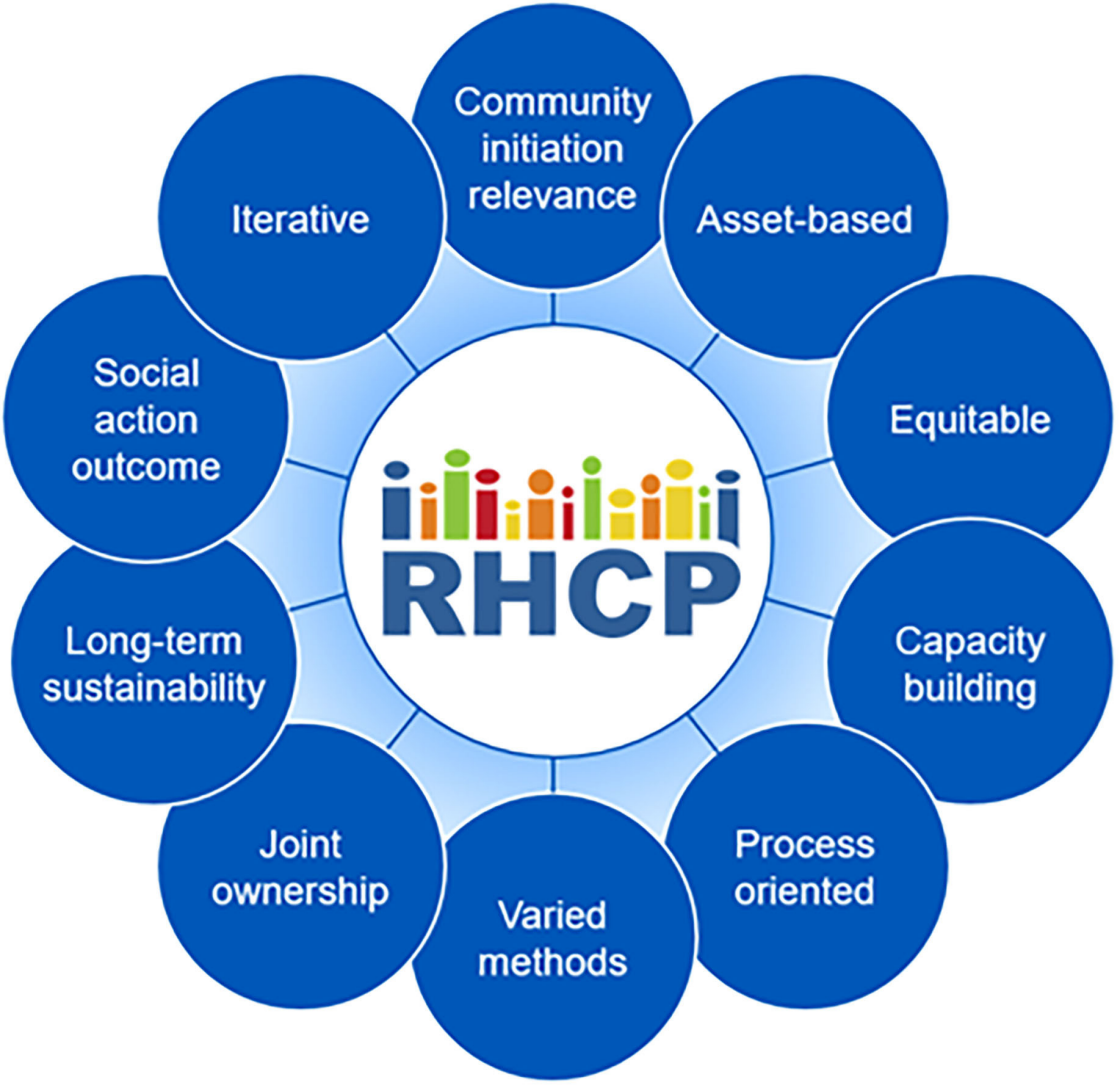
RHCP CBPR principles.

The HEC-Mayo Clinic partnership established connections with the larger community, engaging additional community and academic partners. Partners discussed an ongoing research partnership to address priority health issues of local immigrant and refugee communities. Thus, RHCP was formed. The mission of RHCP is to promote health and wellbeing among the Rochester population through CBPR, education and civic engagement (www.rochesterhealthy.org). In 2007, through a series of meetings and discussions, the partnership matured by formalizing operating norms, adapting CBPR principles (Figure 1), adding dedicated partners from multiple sectors, conducting community health assessments, and discussing potential CBPR projects. Project-specific work group meetings of community and academic partners occur every week and full partnership meetings occur bi-monthly. Community and academic partners conduct every phase of research and programming together and disseminate research results jointly at community forums and academic meetings.

#### Challenges of CBPR partnership initiation

RHCP began in response to a specific community concern and without funding. The lack of funding had benefits and drawbacks. The benefits were that community partners were able to drive the agenda to fully align with health priorities, and the process of partnering without money selected for community and academic partners who were fully dedicated to health equity and authentic community engaged research. The challenges of starting the partnership without funding included a relatively slow pace of work that depended on significant volunteer time and a way of working that may unintentionally exclude community partners with socioeconomic constraints to volunteerism as well as junior faculty members who are under pressure to generate grants and publications at a rapid rate. These challenges were overcome through work with a relatively small coalition initially that focused on building partnership principles and operating norms. This foundation paved the way for a more sustainable partnership once initial funding was secured.

### RHCP then: Moving from project to partnership

#### Identification of broader community health priorities

RHCP has developed an effective community-based and community-led research infrastructure that facilitates extensive research training for partners and deploys data-driven programming among immigrant populations. RHCP first obtained extramural funding from the National Institutes of Health in 2008 for Let's talk about TB to strengthen the CBPR partnership through developing a culturally sensitive health literacy infrastructure for immigrant populations. In 2011, RHCP received funding for Healthy Immigrant Families to test a family-based intervention to preserve dietary quality and physical activity after immigration. This was followed in 2018 with funding for Stories for Change—Diabetes, a digital storytelling intervention to improve diabetes self-management and outcomes among immigrant populations. In 2021, RHCP was awarded funding for Healthy Immigrant Community to foster sustainable health promotion for Southeast Minnesota immigrant communities (Table 1).

#### Co-learning: Community and academic capacity building

Community and academic partners jointly conduct every phase of the research and disseminate research results together at community forums and academic meetings as well as co-authorship on scientific publications and presentations. For community members to fully participate as equal partners and share power over the research process, Mayo Clinic provides training in the protection of research participants, and opportunities for community research capacity building (16). This includes sessions or classes in CBPR, research design, evaluation, and survey implementation. Mayo Clinic sponsors and facilitates formal workshops attended by both community and academic partners in an environment of co-learning. These workshops have included training in CBPR, focus group interviewing and analysis, and digital storytelling (17). Additionally, during the formative stage of the partnership, RHCP organized symposia (2007, 2008) and workshop (2010) attended by both local and national experts in community engagement and community engaged research, bringing communities and researchers together to promote CBPR.

#### Challenges of building a longitudinal CBPR partnership

RHCP's move from a project-focused initiative to a community-wide CBPR coalition met with several challenges. First, new community and academic partners who reached out to RHCP required training on CBPR, basics of study design, ethical conduct of research, etc. Without an infrastructure to support this work, these activities required significant discretionary effort on the part of partners. After learning about the CBPR approach, many potential community partners chose not to participate or disengaged with the work. The primary driver of disengagement early on centered on the tension between research and service. Many community partners and community-based organizations are socialized to partner for the exchange of social services to promote community health, rather than for research. It was imperative for RHCP to be clear that, while social services were often tied into co-creation of interventions, service delivery was not the primary strength or niche of the partnership. It was also important to emphasize that CBPR is not the best approach for all social or health problems. Instead, the focus of RHCP is to rigorously employ research methods through a CBPR approach to impact social change for health equity. This clarity resulted in a smaller coalition of partners than may have been achieved through a broader mission, but it allowed RHCP to thrive through focused action among aligned partners with clear expectations.

Second, the growth of RHCP from project to partnership occurred during a gap in funding that once again challenged the resultant volunteerism of community and academic partners. But, the foundation laid and early partnership successes paved the way for more diverse intramural and then extramural funding opportunities.

Finally, the range of community priorities as identified by the broader RHCP coalition required content expertise beyond the range of the founding academic partners. To meet these needs, RHCP academic partners have systematically engaged content experts from Mayo Clinic and outside academic institutions to fill these gaps. This requires careful onboarding of content experts who are often not used to working in community engaged contexts.

### RHCP now: Lessons learned from sustaining a CBPR partnership

#### Biomedical by name and social by structure

There is an inherent tension that exists between structures of funding and structures of CBPR partnerships. Federal agencies that fund late translational research have become increasingly accepting of participatory approaches to shape intervention development, implementation, and dissemination. Indeed, it is an expectation that investigators describe engagement strategies for community-based and health systems research. However, funding largely remains project-focused rather than partnership-focused. This results in community and academic budgets that target project milestones at the expense of increasing partnership infrastructure needs. Project-specific tasks reflect the biomedical imperative of the intervention (glycemic control, body mass index, etc.,). This model works well for partnerships that are organized around a single study or project. But, for partnerships with multiple concomitant projects, the compounded funding has the potential to strain the partnership's CBPR infrastructure, which is inherently social by structure. Partnership (not project) meetings, community engagement activities, orientation of new community partners and volunteers, CBPR and research trainings, partnership evaluation, and communications (website, social media, etc.,) are examples of longitudinal partnership activities that are vital for partnership health but cannot be funded from protocol-driven budgets. This tension suggests an opportunity for funders and institutions to support the infrastructure to build and sustain partnerships in addition to programmatic support.

#### Integration of diverse learners

While there is growing appreciation for the importance of CBPR and community engagement more broadly, the pathway for training is not self-evident for students and trainees at universities and academic health centers. Learners in public health and healthcare require training to effectively partner with communities to develop and implement strategies that advance health equity and lift up community priorities (19). Best practices for pedagogy around community engagement includes four phases: preparation, action, reflection, and evaluation. These experiences require strong community partners as co-facilitators, a longitudinal trusting relationship between community and academic partners, and careful moderation of reflection/evaluation that centers community context (20). These on-the-ground experiences can be informed or supplemented by existing CBPR curricula (21).

Longitudinal CBPR partnerships like RHCP are uniquely poised to meet these learning needs. RHCP has provided opportunities for more than 500 diverse learners from various disciplines in medicine, nursing, public health, and psychology. For academic partners, these learning opportunities have taken the form of semester-long externships as part of an undergraduate program, month-long research electives for medical students, residents, and fellows, post-doctoral fellowships, and embedded junior faculty experiences. RHCP has also partnered with Winona State University (WSU) for the past 15 years, where undergraduate and graduate nursing students volunteer for RHCP projects under the supervision of WSU faculty who lead reflection and evaluation exercises with their learners. RHCP leaders also ensure that community partners are able to effectively leverage their CBPR experiences to advance their educational pursuits. As an example, four RHCP community partners have gone on to complete medical school and residency training, lending a community-centered lens to their current practice as physicians.

#### Partnership responses to societal contexts

Longitudinal CBPR partnerships are uniquely poised to respond to unexpected shifts in regional and societal contexts that impact health through trusting collaboration between community and academic partners as well as community capacity for evaluation and data collection. The COVID-19 pandemic laid bare societal factors, rooted in structural racism, that resulted in stark racial/ethnic and socioeconomic disparities in outcomes (22, 23). In March 2020, RHCP community partners recognized that reputable COVID-19 information and resources were not reaching immigrant communities with limited English proficiency. RHCP adopted a crisis and emergency risk communication framework to address COVID-19 prevention, testing, and socioeconomic impacts with immigrant and refugee populations in Southern Minnesota. Partners used bidirectional communication between RHCP Communication Leaders and their social networks to refine messages, leverage resources, and advise policy makers. Pilot and implementation studies have demonstrated feasibility, acceptability, reach, 24-month sustainability, scalability and perceived effectiveness of the intervention across multiple groups disproportionately affected by the pandemic (12, 13). The framework was also adapted by longitudinal partnerships in Minnesota, Florida, and Mississippi (24). This model of leveraging longitudinal CBPR partnerships for their trusting relationships with traditionally marginalized communities in a research and evaluation context is a promising approach for centering community voice in response to health crises.

#### Partnership evaluation as a tool for engagement and strategy

**Figure 2:**
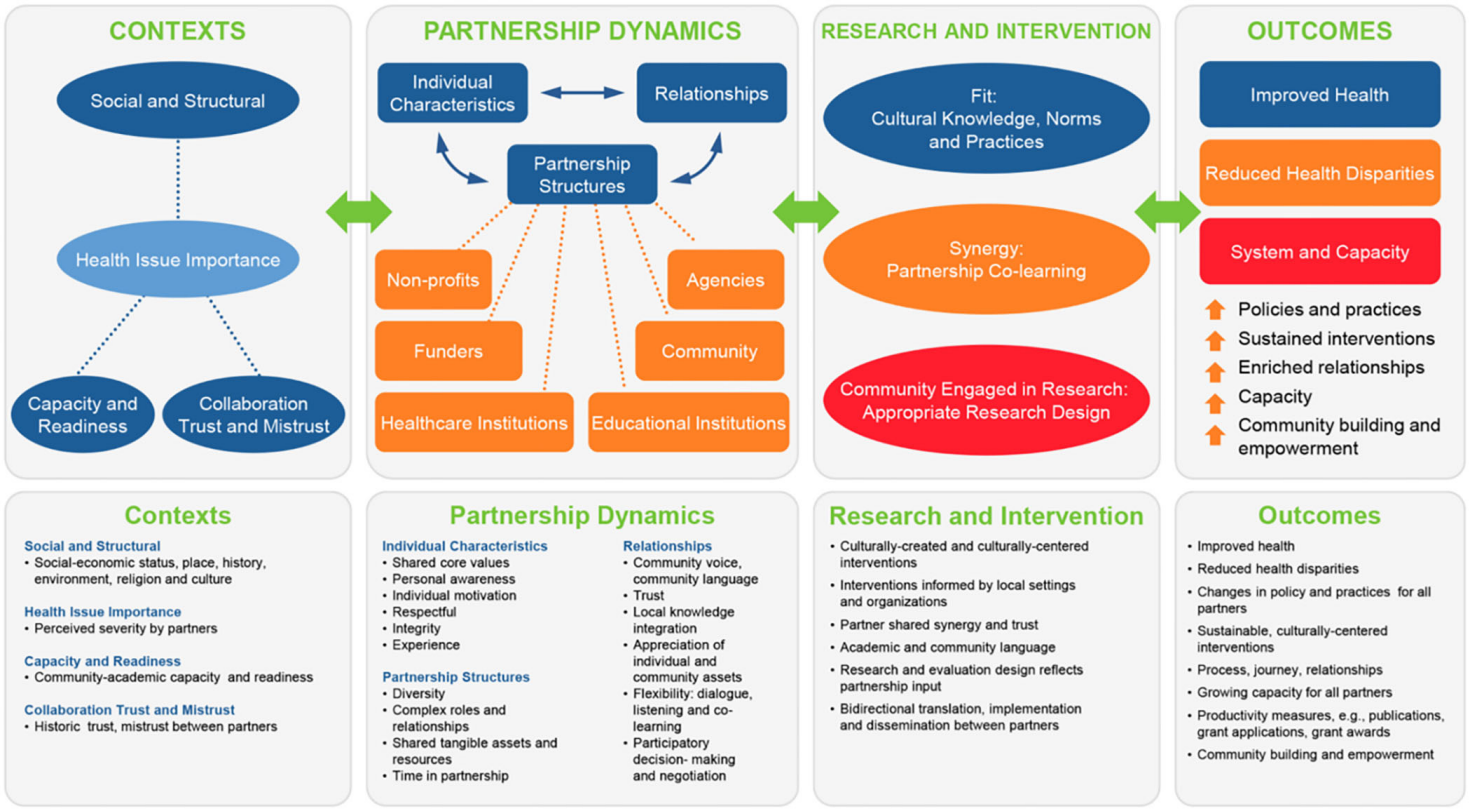
RHCP CBPR conceptual model (18).

Since its inception, RHCP has become a well-established, experienced and productive research partnership, and has included multiple academic and community partners. Over this period of 18 years, RHCP has undertaken a wide-range of health-related projects addressing community-identified health priorities, including those focused on infectious diseases, physical activity and nutrition, diabetes management, and pediatric and adult obesity. During this time, the complexity, breadth and scope of projects also increased, which necessitate increased time and investment from all partners to coordinate and implement projects. Thus, a decade after its inception, RHCP members felt the imperative to revisit the partnership's mission and values and conducted a comprehensive evaluation to determine the overall “health” of the partnership, identify factors that contribute to partnership outcomes, and explore options for sustainability. In 2016, RHCP collaborated with the University of New Mexico Center for Participatory Research for technical assistance, guided by their evaluation tools and empirically-derived CBPR conceptual model (25). The four evaluation steps included: Creation of a partnership timeline; Adaptation of the CBPR conceptual model; Mixed method data collection; and, Participatory data analysis (18). The evaluation showed a high level of trust, a community-driven agenda throughout the research process, and partnership processes that were credited with beneficial RHCP outcomes at the individual, program, community, and policy levels (26). The participatory evaluation analysis enabled partners to explore RHCP's history and contexts, to identify factors that contribute to outcomes, and to plan strategically for the future (26) (Figure 2).

#### Partnership dynamics as barometer and north star

Because CBPR partnerships include long-term, complex relationships between people from different backgrounds, communities, and cultures, trust is an essential ingredient in developing operational guidelines, selecting goals, and conducting research (27). Past research has shown that trust can be facilitated by multi-directional communication and shared decision-making between community partners and academics (28). Trust can be nurtured through the dialogue and reflection essential in a CBPR approach (29). Yet, trust also runs the risk of being fractured by neglecting partnership dynamics (27). Since its inception, RHCP has strategically worked to foster and maintain trusting relationships and used partnership dynamics as a barometer of success. We build trust by opening space for all voices to be heard at meetings, holding group reflections after each event, resolving disputes as they arise, and celebrating our success by sharing meals together. As a result, together we benefit from effective research processes that are culturally appropriate and responsive to the assets and needs of the community.

Beyond relational dynamics, structural dynamics have shaped the long-term progress of RHCP activities, including shared assets and resources as well as long-term commitments from partners. Recent studies have underscored the compounding importance of structural governance and collective empowerment (30, 31). RHCP addresses structural governance through adherence to shared CBPR principles, operating norms, and its CBPR conceptual model in order to ensure that community priorities guide the research agenda, which is evident by the wide range of research topics undertaken by the partnership. RHCP partners have explored the possibility of becoming a legal entity [e.g., 501(c) (3) organization], but have decided against this structure due to the additional infrastructure burdens it would impose. However, this decision results in a potential missed opportunity for more formal governance structures to codify its values and ways of working, which have been important mechanisms of effective structural governance in other contexts (32).

#### Lessons learned for sustaining a CBPR partnership

RHCP community and academic partners have learned many lessons on sustaining a CBPR partnership over the last 18 years. Most of these lessons have been born of finding ways to overcome key and frequently faced challenges. First, community-based organizations and advocacy groups engaged in CBPR work are often small, with limited administrative infrastructure, budget and personnel. Yet, these small groups can reflect community voice with grassroots authenticity that is more difficult to emulate in larger organizations. This lack of community infrastructure can be a barrier to consistent engagement, even when these projects are relevant to the communities they serve. Similarly, rapid transitions in leadership and personnel among partnering organizations can lead to a change in partnership relational dynamics, affecting both ongoing and future engagement in CBPR. To overcome these challenges, flexibility in project timelines, both in processes and outcomes, is important, while actively seeking to identify upcoming challenges and brainstorming solutions together. This includes intentional agreement about meeting times and forecasting of competing priorities, holidays, and community events that may impact project timelines. Furthermore, succession planning, meeting alternates, having more than one key person in a community organization, and structural governance help to ensure continuity and reduce the risk of fracturing longitudinal relationships.

RHCP has also experienced challenges to sustainability of academic partners. Despite the rapid growth of CBPR approaches in the US (33), investigators with interest in CBPR often do not appreciate the investment in time (often in “off” hours) and relationship building. For those who are accustomed to traditional research approaches, this focus and commitment can seem time consuming, with results long in coming. In our experience, despite a strong initial interest in becoming an RHCP academic partner, only a relatively few invest in making this a large component of their careers. More intensive investment at the partnership level and institutional level in the small number of investigators dedicated to making CBPR the foundation of their career may be more fruitful than investing in loose ties to CBPR in academic settings.

As noted above, funding is often project based, leaving no specific support for administrative and partnership infrastructure. Frank and open discussions among all partners around funding and finances is critical for trust-building and sustainability. Commitment by partners in RHCP has ensured that engagement continues throughout, including during “dry spells” when there is limited funding, taking these opportunities to continue with capacity building for both community and academic partners. This commitment is often a testament to the level of investment both community and academic partners have to their communities and the mission of RHCP. Furthermore, advocacy is needed to ensure institutional support of CBPR infrastructure to promote sustainability.

## Conclusions

RHCP is an 18-year CBPR partnership that works to address issues of health promotion among immigrant and refugee populations in Southeast Minnesota with extended networks throughout the US. The partnership has taken a circuitous research agenda that reflects community priorities and capacities with shared values informed by its CBPR principles, operating norms, and conceptual model. Challenges are encompassed by the tension between project-based funding and the needs of a social, longitudinal infrastructure that transcends individual projects. Long-term translation of partnership successes have resulted in sustainable, community-led change.

## Data availability statement

The original contributions presented in the study are included in the article/supplementary material, further inquiries can be directed to the corresponding author.

## Author contributions

All authors listed have made a substantial, direct, and intellectual contribution to the work and approved it for publication.
